# Supplementary material for: Automated single-cell proteomics providing sufficient proteome depth to study complex biology beyond cell type classifications
Source: Nat Commun. 2024 Jul 8;15:5707. doi: 10.1038/s41467-024-49651-w (PMC11231172; doi:10.1038/s41467-024-49651-w)
Supplement: Supplementary file 1 — Supplementary Information [file 41467_2024_49651_MOESM1_ESM.docx]

# Title

Automated single-cell proteomics providing sufficient proteome depth to study complex biology beyond cell type classifications

# Authors

Claudia Ctortecka^1^, Natalie M. Clark^1^, Brian W. Boyle^1^, Anjali Seth^2^, D. R. Mani^1^, Namrata D. Udeshi^1^ & Steven A. Carr^1,*^

# Affiliation

1. Broad Institute of MIT and Harvard, 415 Main Street, 02142 Cambridge, MA, USA.
2. Cellenion SASU, 60F avenue Rockefeller, 69008 Lyon, France.

# Correspondence

Claudia Ctortecka

cctortec@broadinstitute.org

Steven A. Carr

scarr@broad.mit.edu

Broad Institute of MIT and Harvard

415 Main Street

02142 Cambridge

MA, USA.


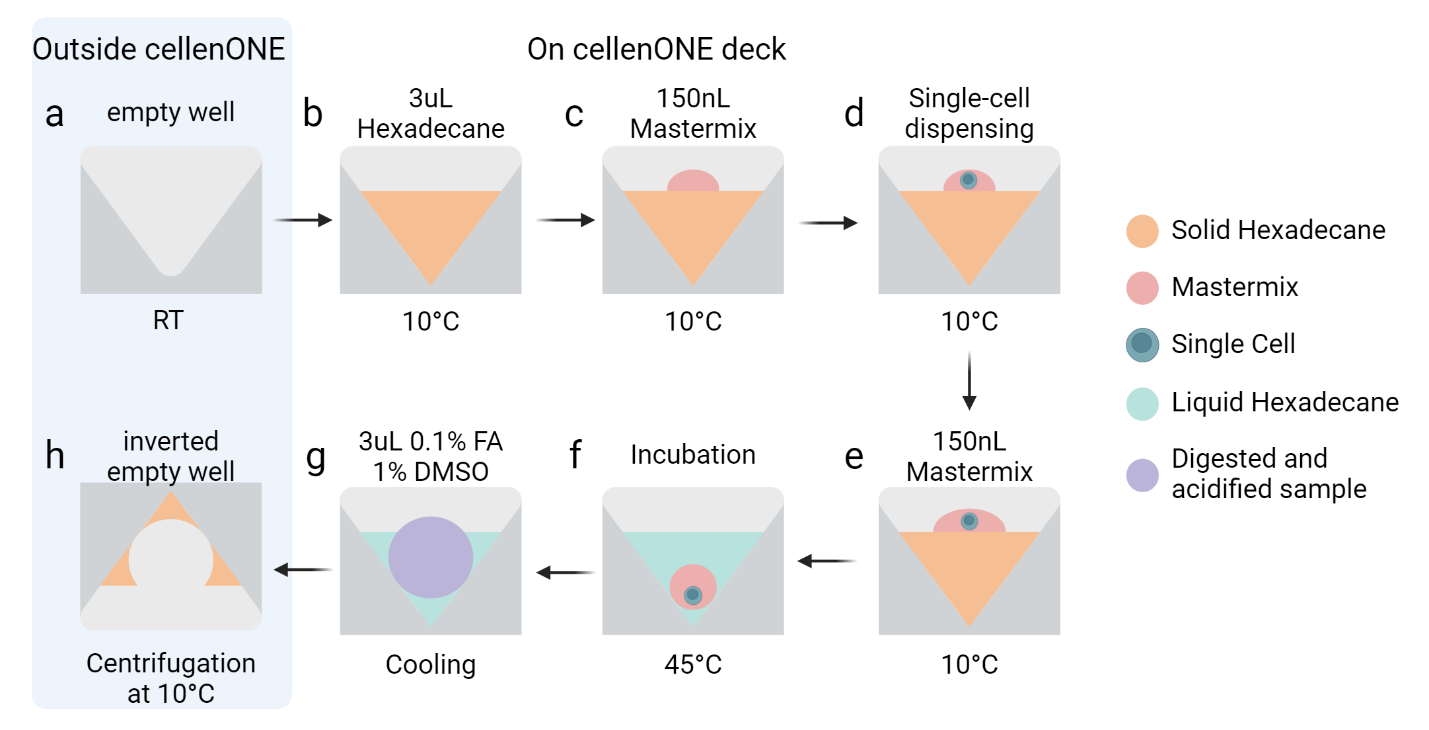


Supplemental Fig. 1: Illustration of (a) an empty proteoCHIP EVO*96 nanowell. Prior to insertion into the cellenONE, each well is filled with 3 µL hexadecane. (b) After the chip is inserted into the cellenONE and the temperature is dropped to 10°C the hexadecane solidifies. (c) On top of the solidified hexadecane 150 nL of the mastermix is dispensed, (d) followed by addition of a single cell matching the isolation criteria. (e) On top of the still solidified hexadecane another 150 nL of mastermix is dispensed to ensure that the single cell is submerged. (f) Following increasing of the temperature to 45°C the hexadecane melts, submerging the sample droplet and the single cell to overome evaporation during the 2 hr lysis and digestion. (g) After incubation and continuous rehydration at 45°C the sample droplet volume is increased to 3 µL with 0.1% FA and 1% DMSO. (h) After decreasing the temperature of the proteoCHIP again to 10°C the hexadecane solidifies. The chip is inverted on top of the conditioned Evotips. The now exposed sample droplet is then transferred to the Evotips through centrifugation, leaving the solidified hexadecane in the proteoCHIP EVO*96 well. Created with BioRender.com, released under a Creative Commons Attribution-NonCommercial-NoDerivs 4.0 International license.


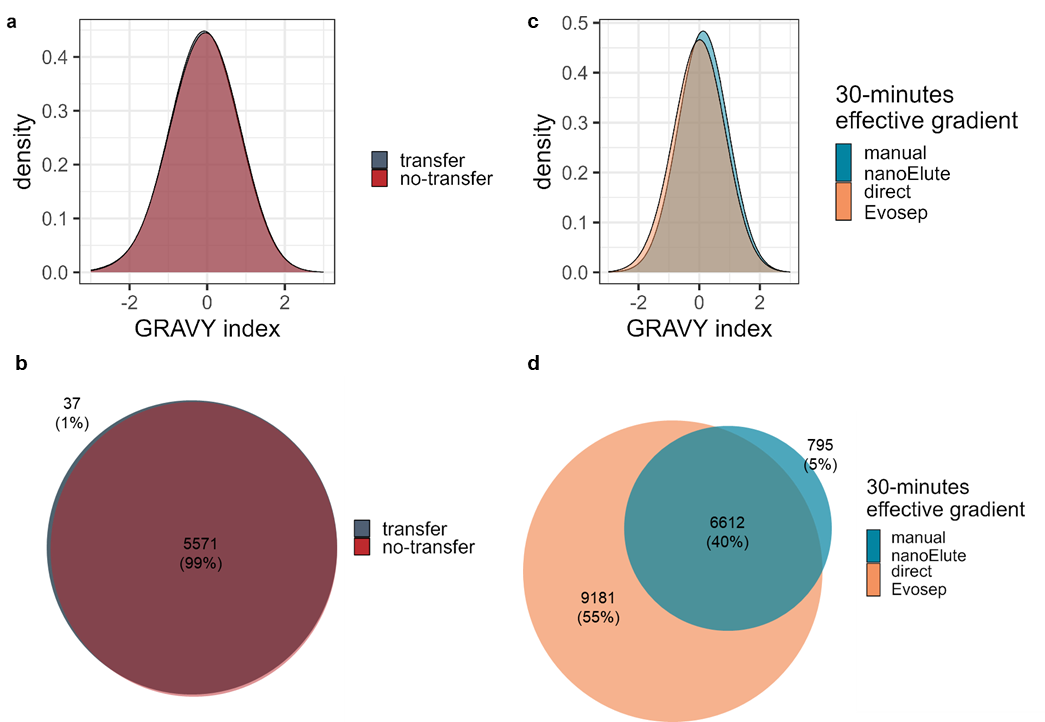


Supplemental Fig. 2: (a) GRAVY index of peptides identified in ddaPASEF with manual transfer (gray; n = 5) and automated transfer via centrifugation (red; n = 5) and (b) their unique peptide sequence overlap. (c) GRAVY index of peptides identified in diaPASEF with 30-minutes effective gradients on the nanoElute with manual transfer to a HPLC vial (dark green; n = 25) or automated transfer to the Evotip and acquisition with 40SPD (orange; n = 25) and (d) their unique peptide sequence overlap.


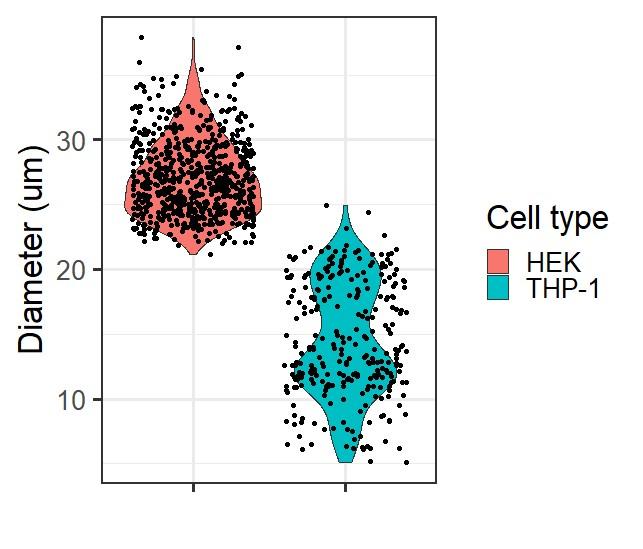


Supplemental Fig. 3: Density distribution of dispensed HEK-293T (n = 672) and THP-1 (n = 288) cell diameters. Boxplot center line indicates the median, the box limits represent the upper and lower quartiles, and the whiskers represent 1.5x the interquartile range. 'Source data are provided as a Source Data file.'


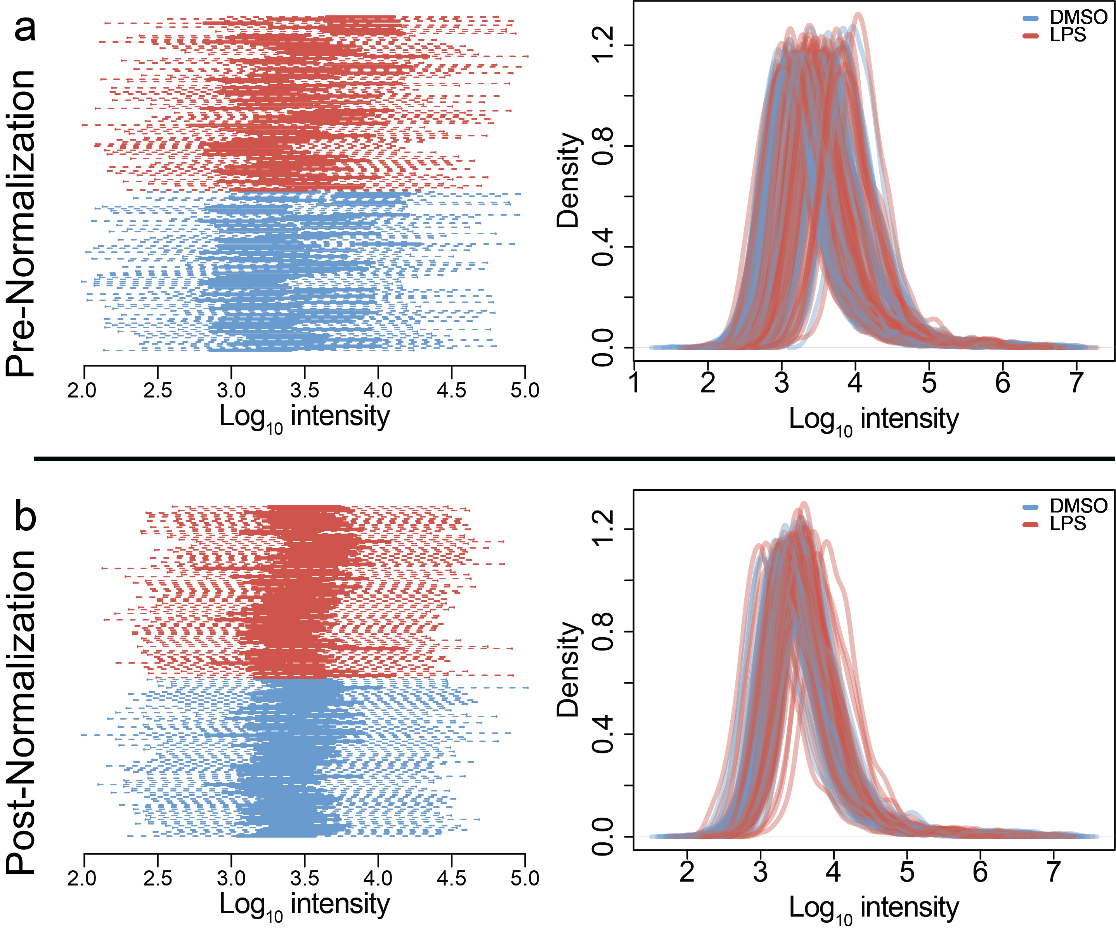


Supplemental Fig. 4: Boxplots (a) and profile plots (b) of log_10_-transformed protein intensities before (top) and after (bottom) normalization using SCnorm. Each boxplot/density represents the distribution of proteins from one cell. Cells are colored by LPS treatment. Boxplot center line indicates the median, the box limits represent the upper and lower quartiles, and the whiskers represent 1.5x the interquartile range. 'Source data are provided as a Source Data file.'


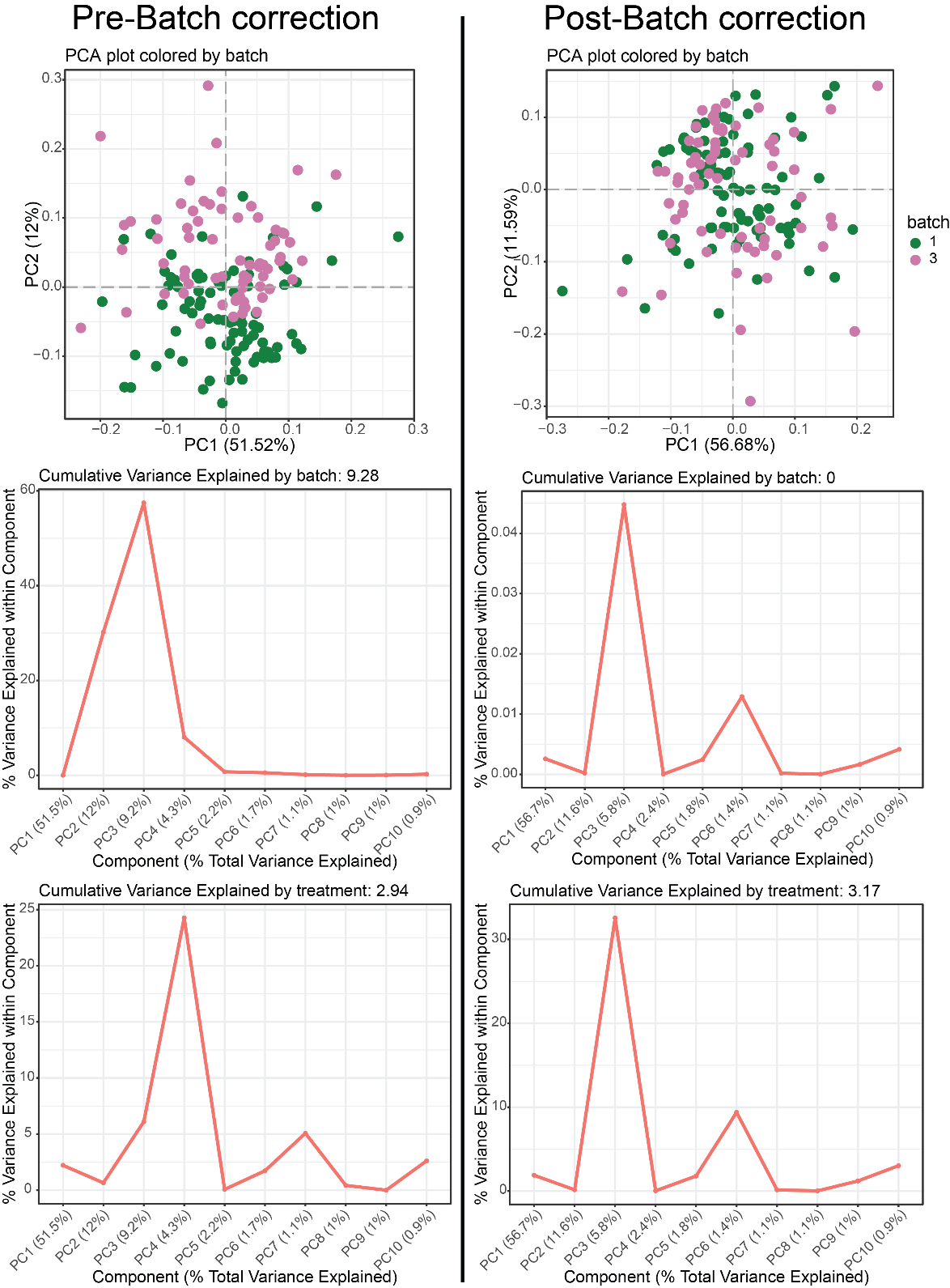


Supplemental Fig. 5: PCA and PC regression of LPS treated (n = 84) with DMSO control (n = 77) THP-1 cells (a) pre- and (b) post batch correction. PC regressions indicates cumulative variance explained by batch or group (= treatment). Colors indicate experimental batches. 'Source data are provided as a Source Data file.'


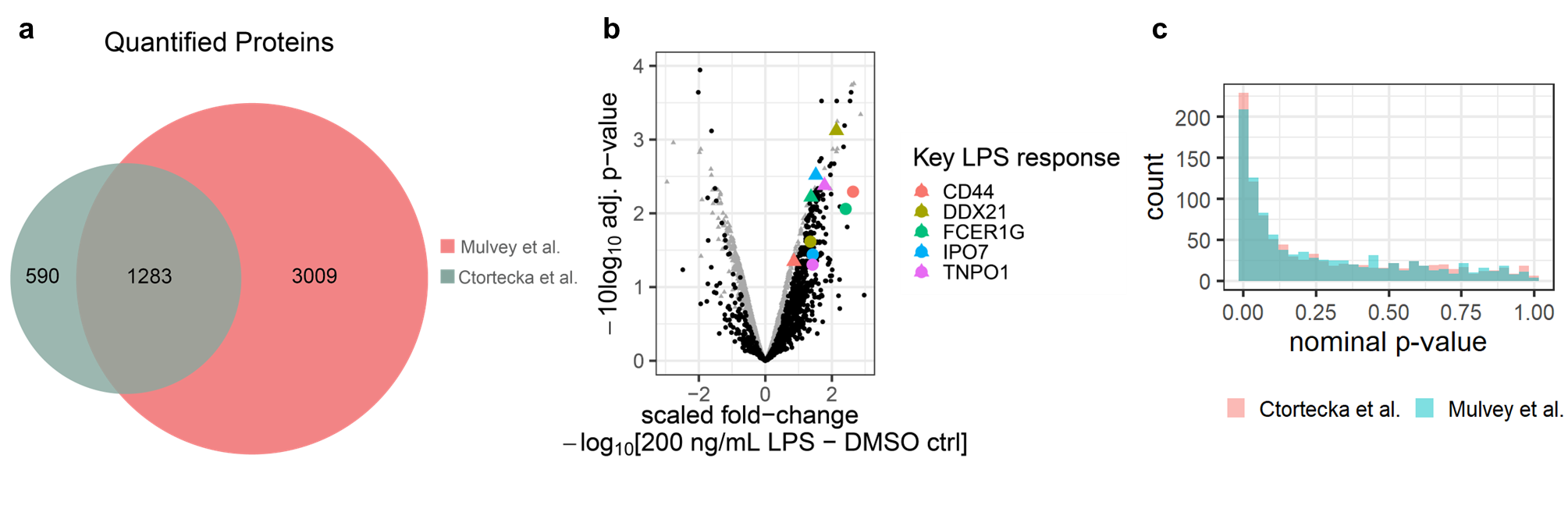


Supplemental Fig. 6: (a) Overlap of all quantified proteins of previously published bulk LPS response proteins in THP-1 cells (pink) and single THP-1 cells acquired for the presented study (green; n = 161) upon 12 hours LPS treatment. (b) Volcano plot of two-sided, two-sample *t-*test results (200 ng/mL LPS over DMSO vehicle control) presented in this study (black dots) and from Mulvey et al., (grey triangle). Log_10_ fold change and -10log_10_ p-value are shown, significantly regulated key LPS proteins with an adjusted p-value ≤0.05 are colored with the respective symbol (Dot = this study, triangle = Mulvey et al.). (c) Binned distribution of nominal p-values of proteins overlapping between this study and Mulvey et al. 'Source data are provided as a Source Data file.'
