# Supplementary material for: Automated single-cell proteomics providing sufficient proteome depth to study complex biology beyond cell type classifications
Source: Nat Commun. 2024 Jul 8;15:5707. doi: 10.1038/s41467-024-49651-w (PMC11231172; doi:10.1038/s41467-024-49651-w)
Supplement: Supplementary file 4 — Description of Additional Supplementary Files [file 41467_2024_49651_MOESM4_ESM.pdf]

File Name: Supplementary Data 1
Description: ProTIGY v1.1.7. exports of batch corrected exports of normalized single cell data including the experimental design and sample annotations. A moderated two-sided, two sample t-test was performed without adjustments for multiple comparisons.

File Name: Supplementary Data 2
Description: Gene Set Enrichment Analysis (GSEA) on the signed $\log_{10}$ p-values from the two sided, two-sample t-test using ssGSEA 2.0 (https://github.com/broadinstitute/ssGSEA2.0) on the Reactome gene sets (https://www.gsea-msigdb.org/gsea/msigdb/human/genesets.jsp?collection=CP:REACTOME)

File Name: Supplementary Data 3
Description: Detailed diaPASEF isolation window placement

File Name: Supplementary Data 4
Description: Detailed isolation parameters for HEK293 and THP-1 cells acquired during cellenONE based cell isolation.
